# CD33 competitive inhibitor peptide IMAD‐001 modulates innate immunity in Alzheimer’s disease

**DOI:** 10.1002/alz.089331

**Published:** 2025-01-09

**Authors:** Jennifer L Green, Dallin Dressman, Mamun Rashid, Ryan Hobson, Wassim Elyaman, Elizabeth M Bradshaw

**Affiliations:** ^1^ Columbia University Irving Medical Center, New York, NY USA; ^2^ Department of Neurology, Columbia University, New York, NY USA; ^3^ Columbia University Medical Center, New York, NY USA; ^4^ Taub Institute for Research on Alzheimer’s Disease and the Aging Brain, Columbia University, New York, NY USA

## Abstract

**Background:**

Genetic studies indicate a causal role for microglia, the innate immune cells of the central nervous system (CNS), in Alzheimer’s disease (AD). Despite the progress made in identifying genetic risk factors, such as CD33, and underlying molecular changes, there are currently limited treatment options for AD. Based on the immune‐inhibitory function of CD33, we hypothesize that inhibition of CD33 activation may reverse microglial suppression and restore their ability to resolve inflammatory processes and mitigate pathogenic amyloid plaques, which may be neuroprotective. Here, we investigate the effects of a competitive inhibitor peptide of CD33 binding, IMAD‐001, on downstream immune response in AD.

**Method:**

Primary human monocyte‐derived microglia‐like cells (MDMi) and monocytes were treated with IMAD‐001 and we determined its effect on microglial function and immune signaling by measuring gene expression changes in markers of microglial activation by qPCR and amyloid beta (Aβ) uptake by flow cytometry. To measure the *in vivo* effects, we treated 12‐month‐old 5xFAD mice with weekly subcutaneous injections of 5mg/kg IMAD‐001 or vehicle control for 28 days and examined Aβ clearance by immunohistochemistry and CNS immune cell phenotypes by single‐cell (sc)RNA‐seq.

**Result:**

IMAD‐001 treatment increased Aβ uptake in primary monocytes. In *in vivo* experiments, we see clearance of Aβ in the brains of the 5xFAD mice with weekly administration. Using scRNA‐seq of CD45+ cells, we identified the expected populations in the brains of the animals and found that peptide treated mice had an increase in classical dendritic cells and a slight decrease in disease‐associated microglia (DAM) and homeostatic microglia. Microglia cells regulated their phenotype in response to the peptide as well with notable downregulation of CD274/PD‐L1, an immunosuppressive molecule in which disruption of its interaction with PD‐1 was found to clear Aβ and improve cognitive performance. Interestingly, peptide treatment resulted in downregulation of IFITM3 and upregulation of CD74, suggesting CD33 inhibition influences microglia to inhibit Aβ production.

**Conclusion:**

IMAD‐001 inhibition of CD33 resolved Aβ induced suppressed microglial immune signaling and increased microglial phagocytosis and response to their environment *in vitro* and *in vivo*, without inducing inappropriate activation, suggesting its potential for therapeutic use in AD.

